# Causal relationship between gut microbiota and glioblastoma: a two-sample Mendelian randomization study

**DOI:** 10.7150/jca.90149

**Published:** 2024-01-01

**Authors:** Chao Ju, Yanjing Chen, Longtao Yang, Yijie Huang, Jun Liu

**Affiliations:** 1 Department of Radiology, The Second Affiliated Hospital of Xinjiang Medical University, Urumqi, 830011, China.; 2 Department of Radiology, The Second Xiangya Hospital of Central South University, Changsha, Hunan, 410011, China.

**Keywords:** Gut microbiota, glioblastoma, Mendelian randomization, Genetics, SNPs

## Abstract

**Background:** Observational research and medical trials have suggested a connection between gut microbiota and glioblastoma, but it remains unclear if the relationship is causal.

**Method:** A two-sample Mendelian randomization (MR) study was conducted by employing data from the MiBioGen consortium's largest genome-wide association study (n=18340) and the FinnGen consortium R8 release information (162 cases and 256,583 controls). Inverse variance weighted (IVW), weighted median estimator (WME), weighted model, MR-Egger, simple mode, and MR-PRESSO were used to determine the causal relationship between gut microbiota and glioblastoma. Reverse MR analysis was also performed on bacteria identified as causally related to glioblastoma.

**Results:** Seven causal relationships were identified between genetic liability in the gut microbiota and glioblastoma, involving various bacterial families and genera. No significant causal effect was found on gut microbiota from glioblastoma, and no significant heterogeneity of instrumental variables (IVs) or horizontal pleiotropy was observed.

**Conclusion:** A two-sample MR analysis reveals a causal association between the gut microbiota and glioblastoma, highlighting the need for more investigation to comprehend the processes behind this association.

## Introduction

Gut microbiota including bacteria, viruses, and fungi refers to the collection of microorganisms, which reside in the human gastrointestinal tract 1. They play an essential role in various physiological functions, such as digestion, nutrient absorption, and immune system regulation 2. Recent studies have shown that gut microbiota is also associated with the risk of several diseases, including metabolic diseases 3, autoimmune diseases 4, rheumatoid arthritis 5 and cancer 6,7.

Glioblastoma is a highly aggressive brain tumor that arises from glial cells in the brain. It is one of the most common and deadly forms of primary brain cancer, with less than 10% five-year survival rate, with a median survival time of less than 15 months 8. The current treatment options for glioblastoma include radiation therapy, surgery, and chemotherapy, but their efficacy is limited and the prognosis remains poor 9.

Bidirectional communication system “gut-brain axis” connects gastrointestinal tract and central nervous system 10. Studies have shown that gut microbiota dysbiosis, or an imbalance in gut microbial community, can lead to the development of various cancers, including brain tumors 11. In addition, preclinical studies demonstrated that certain gut bacteria could modulate immune system and affect the efficacy of cancer therapies, including those used to treat glioblastoma 12.

MiBioGen is a comprehensive database of microbial genomes, which provides a platform for ours to access and analyze microbial genomic data. The database is designed to help ours understand the genetic basis of microbial evolution, ecology, and pathogenesis 1. MiBioGen contains a vast collection of bacterial and archaeal genomes, as well as tools for comparative genomic analysis, annotation, and visualization 13,14. The FinnGen study is a large-scale genetic study of various disorders in the Finnish population. The study aims to identify genetic factors that contribute to risk of disorders, as well as to understand the biological mechanisms underlying the disorders 14. The FinnGen database contains genomic data from over 160,000 individuals, including both cases and controls. The data includes information on genetic variants, as well as clinical and phenotypic data 15.

MR is a popular statistical technique used in observational studies to estimate causal effect of an exposure on an outcome by leveraging genetic variation as IVs 16. This approach exploits random allocation of genetic variants at conception to determine impact of an exposure on an outcome of interest 17. Two-sample MR approach involves using separate datasets for the genetic variants and the exposure-outcome data, which allows for increased statistical power and flexibility in the analysis 18,19. One of the key advantages of two-sample MR is that it enables researchers to estimate causal effects for a wide range of exposures and outcomes, without the need for expensive or time-consuming data collection 20. Additionally, it can help to overcome some limitations of traditional MR, such as weak instrument bias and pleiotropy (when a single genetic variant influences multiple traits) 13. Overall, Two-sample MR is a powerful and flexible approach that can provide valuable insights into causal relationships between exposures and outcomes.

Therefore, we used the MibioGen and FinnGen databases for the first time to investigate causal relationship between gut microbiota and glioblastoma using two-sample MR method, eventually to accelerate the pace of discovery in the field of human genetics, and provide new insights into genetic basis for disease.

## Material and Methods

### Study design

**Figure 1:**
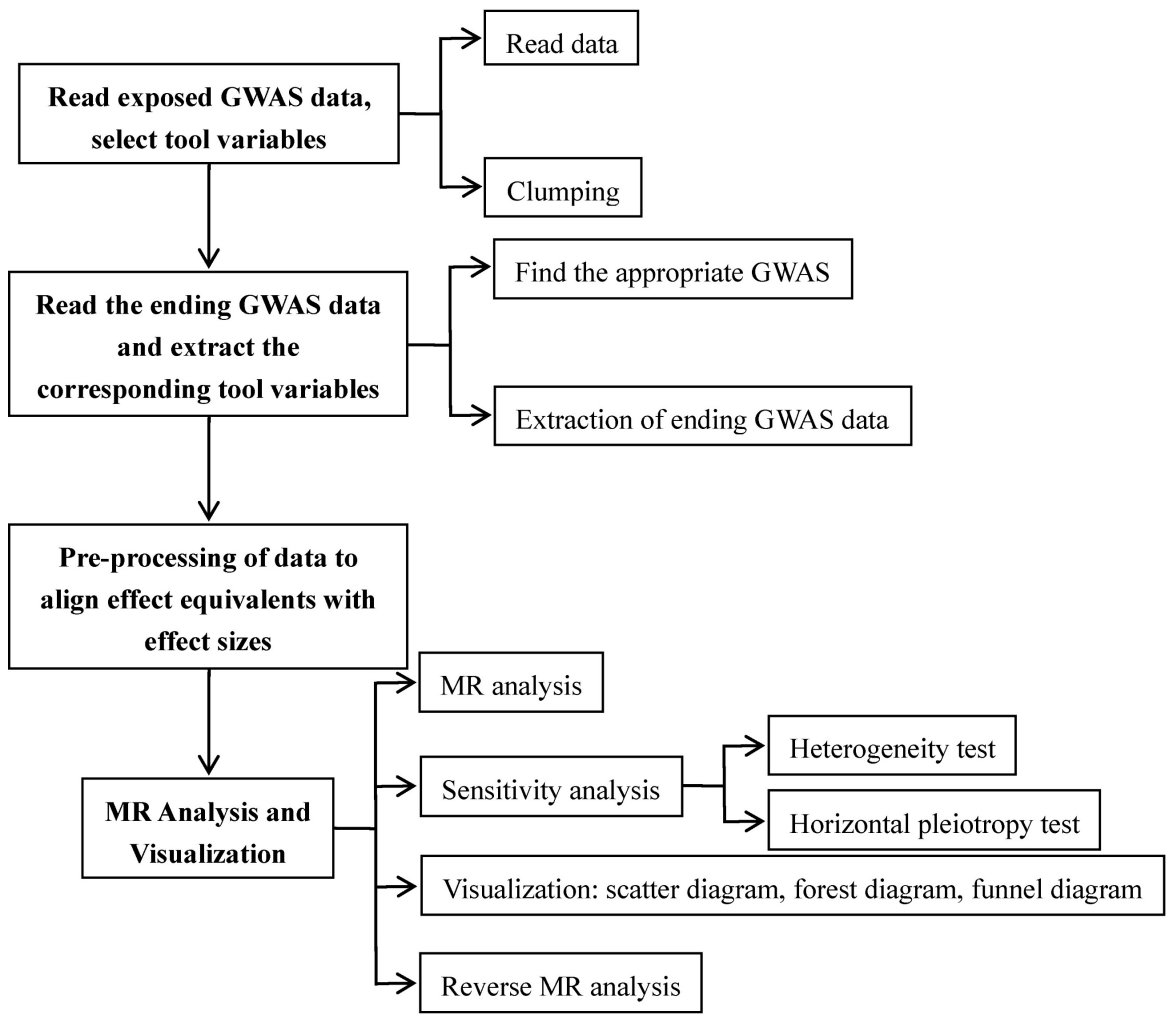
Study design and workflow

Using a two-sample MR methodology, we assessed the link between the gut microbiota and glioblastoma. To thoroughly study the role played by gut microbiota in the etiology of glioblastoma, we conducted MR studies at five distinct character levels, including phylum, class, order, family, and genus. **Figure 1** depicts the research design as well as the fundamental MR assumptions.

### Exposure data

The aim of the study was to investigate correlations between human genetic variation and gut microbiota, specifically through the use of SNPs linked to composition of human gut microbiota as IVs in a GWAS dataset. The International Consortium MiBioGen conducted a large-scale multi-ethnic GWAS, which analyzed genotyping data and 16S ribosomal RNA gene sequencing from a total of 18,340 participants across 24 cohorts from various countries, including the Germany, United States, Denmark, Canada, Israel, Finland, the United Kingdom, the Netherlands, Belgium, Sweden, and Korea 13,21. The study identified 211 taxa, including 9 phyla, 16 classes, 20 orders, 35 families, 131 genera 1.

### Outcome data

The FinnGen consortium R8 release data 14,22 provided the GWAS summary statistics for glioblastoma. The GWAS involved 256,745 Finnish individuals, of which 162 were cases and 256,583 were controls. To ensure accuracy, the analysis accounted for sex, age, first 10 principal components, and genotyping batch 14.

### Instrumental variable selection

IVs is an abbreviation for instrumental variables. The MR method employs genetic variants as IVs to infer the causality of an association. The IVs were selected based on the following criteria: (1) potential IVs were single nucleotide polymorphisms (SNPs) associated with each taxa at the locus-wide significance threshold (P < 5.0 × 10^-6^) 3; (2) the linkage disequilibrium (LD) between the SNPs was calculated using the 1000 Genomes project European samples data as the reference panel, and among those SNPs with R^2^ < 0.001 (clumping window size=10,000 kb), only the SNPs with the lowest P-values were retained; (3) SNPs that have a minor allele frequency (MAF) of ≤ 0.01 were removed; and (4) in the presence of palindromic SNPs existed, forward strand alleles were inferred using allele frequency information.

### Statistical analysis

**Figure 2:**
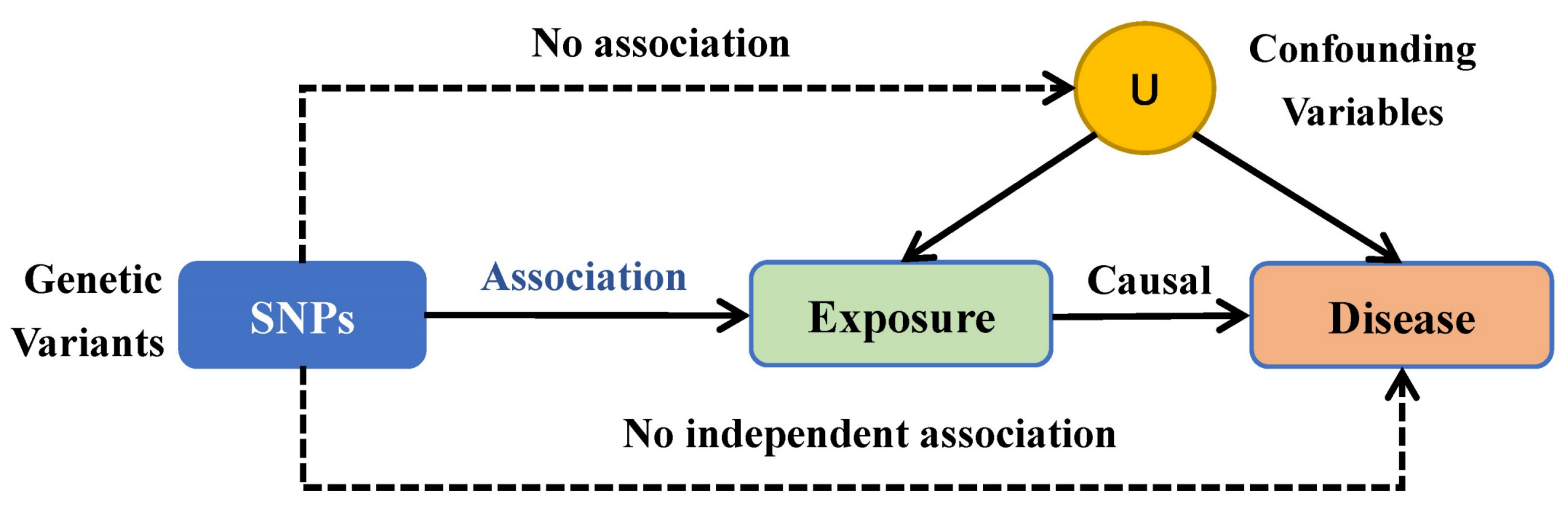
Mendelian randomization (MR) methods

We conducted a study for examining the relationship between features of the microbiome and glioblastoma by employing MR analysis **(Figure 2)**. For features with different IVs, we used six popular MR methods 23, including IVW 20, weighted mode 24, simple mode 24, MR-Egger regression 25, WME 26, and MR-PRESSO 27. The IVW approach is mentioned to be barely extra effective than the others underneath sure stipulations 26. Therefore, the consequences with extra than one IV have been usually primarily based on the IVW method, with the other different five methods serving as complements 28.

Three major principles of MR method selection 29: (1) Preferential use of IVW estimates in the absence of heterogeneity and multi-effects; (2) When there is only heterogeneity and no multi-effects, the results of the WME method are used in preference (the random effects model of IVW can also be used); (3) When there is multiplicity of effects, the results calculated by MR-Egger method are used in preference.

Leave-one-out method refers to gradually eliminate each SNP, calculating meta-effects of remaining SNPs, and observing whether results change after eliminating each SNP, if the results change significantly after eliminating a certain SNP, it means that the presence of a certain SNP has a significant impact on the results 30.

One crucial issue in MR studies is the presence of weak instrumental variable bias 31. From a traditional empirical perspective, when the F-statistic is blow 10, we typically consider genetic variants as weak instrumental variables. This may introduce some bias into the results, and therefore caution should be exercised in interpreting them at this stage. Ideally, an F-statistic greater than 10 or even greater than 100 would be preferred 32.

Heterogeneity tests were conducted using both Cochran's Q statistic and a two-sample MR package across instruments. Evidence of heterogeneity and invalid instruments was indicated by Q values greater than the number of instruments minus one, while Q statistic values significant at p-values < 0.05 suggested the presence of heterogeneity 33,34.

In order to investigate whether glioblastoma has any causal influence on the noteworthy bacterial, we conducted a reverse MR analysis. In this analysis, glioblastoma was considered as exposure, and identified causal bacterial was treated as outcome. To accomplish this, we employed SNPs associated with glioblastoma as IVs 30. The settings and procedures used were in line with forward MR.

We performed all statistical analyses using R version 4.2.2 (R Foundation for Statistical Computing, Vienna, Austria). MR analyses had been carried out the usage of the Two-sample MR (version 0.5.6) 35, MR-PRESSO (version 1.0) 27, and qvalue (version 2.30.0) 36 R packages.

## Results

### SNP selection

Initially, we detected 65, 120, 150, 260, and 902 SNPs related to gut microbiota at the phylum, class, order, family, and genus levels, correspondingly, at a p-value threshold of less than 5 × 10^-6^. Following several quality control procedures, we handpicked 35 SNPs as instrumental variables (IVs) that met genome-wide statistical significance threshold of p < 5 × 10^-6^ **(Table S1)**.

All IVs' *F* statistics exceeded 10, implying the absence of weak instrument bias. Furthermore, MR-PRESSO global test found no evidence of pleiotropic effects (p > 0.05). Finally, after discarding pleiotropic SNPs flagged by MR-PRESSO outlier test and MR-Egger regression, no signs of horizontal pleiotropy were observed in IVs (both MR-PRESSO global test and MR-Egger regression yielded p-values greater than 0.05).

### Causal effects of gut microbiota on the development of glioblastoma

**Figure 3:**
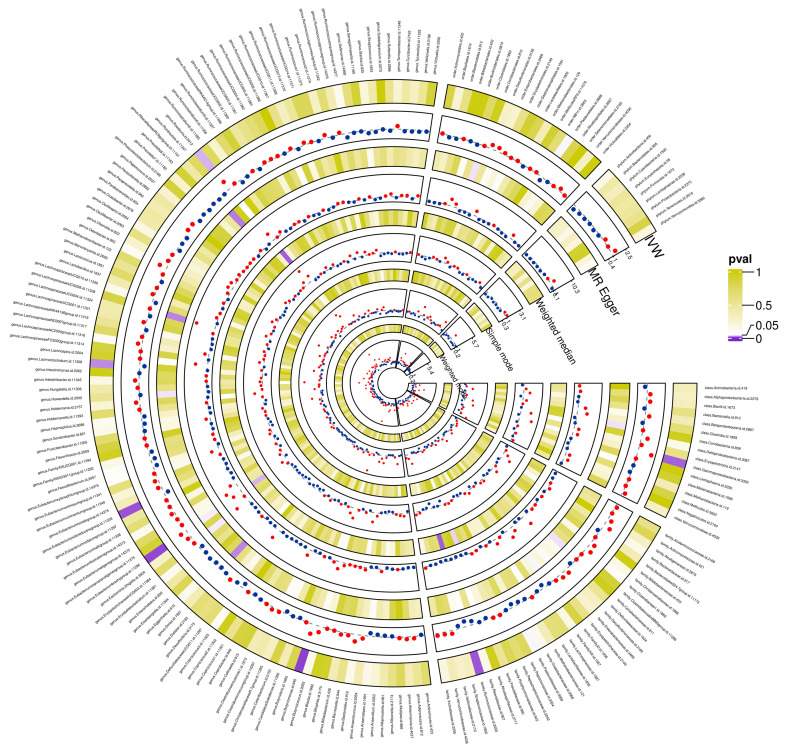
Preliminary correlations between gut microbiota and glioblastoma determined from five popular MR methods. P < 0.05 values were displayed in purple, whereas P > 0.05 estimates were displayed in white or yellow.

The preliminary associations between bacterial clusters of various levels of classification derived from five popular MR methods and glioblastoma was presented in **Figure 3**. In the IVs dataset (p < 5 × 10^-6^), we found a causal relationship between three microbial families and four microbial genera of the gut microbiota and glioblastoma **(Table 1)**. Among the three microbial families, family Ruminococcaceae (OR = 0.094, 95% CI = 0.021-0.417, p = 0.19 × 10^-2^) was shown to be protective against glioblastoma as assessed by IVW, whereas an increase in the other two microbial families, Bacteroidaceae (OR = 12.003, 95% CI = 1.793-80.32, p = 1.03 × 10^-2^) and Peptococcaceae (OR = 3.656, 95% CI = 1.233-10.841, p = 1.94 × 10^-2^), were associated with a high risk of glioblastoma development. In addition, an increase in four microbial genera, Eubacterium (brachy group) (OR = 4.431, 95% CI = 1.529-12.842, p = 0.61 × 10^-2^), Actinomyces (OR = 18.805, 95% CI = 2.116-167.165, p = 0.85 × 10^-2^), Bacteroides (OR = 12.003, 95% CI = 1.794-80.320, p = 1.04 × 10^-2^) and Ruminiclostridium6 (OR = 3.641, 95% CI = 1.009-13.139, p = 4.84 × 10^-2^) were found to be associated with an increased risk of glioblastoma as assessed by IVW.

### Sensitivity analyses

**Figure 4:**
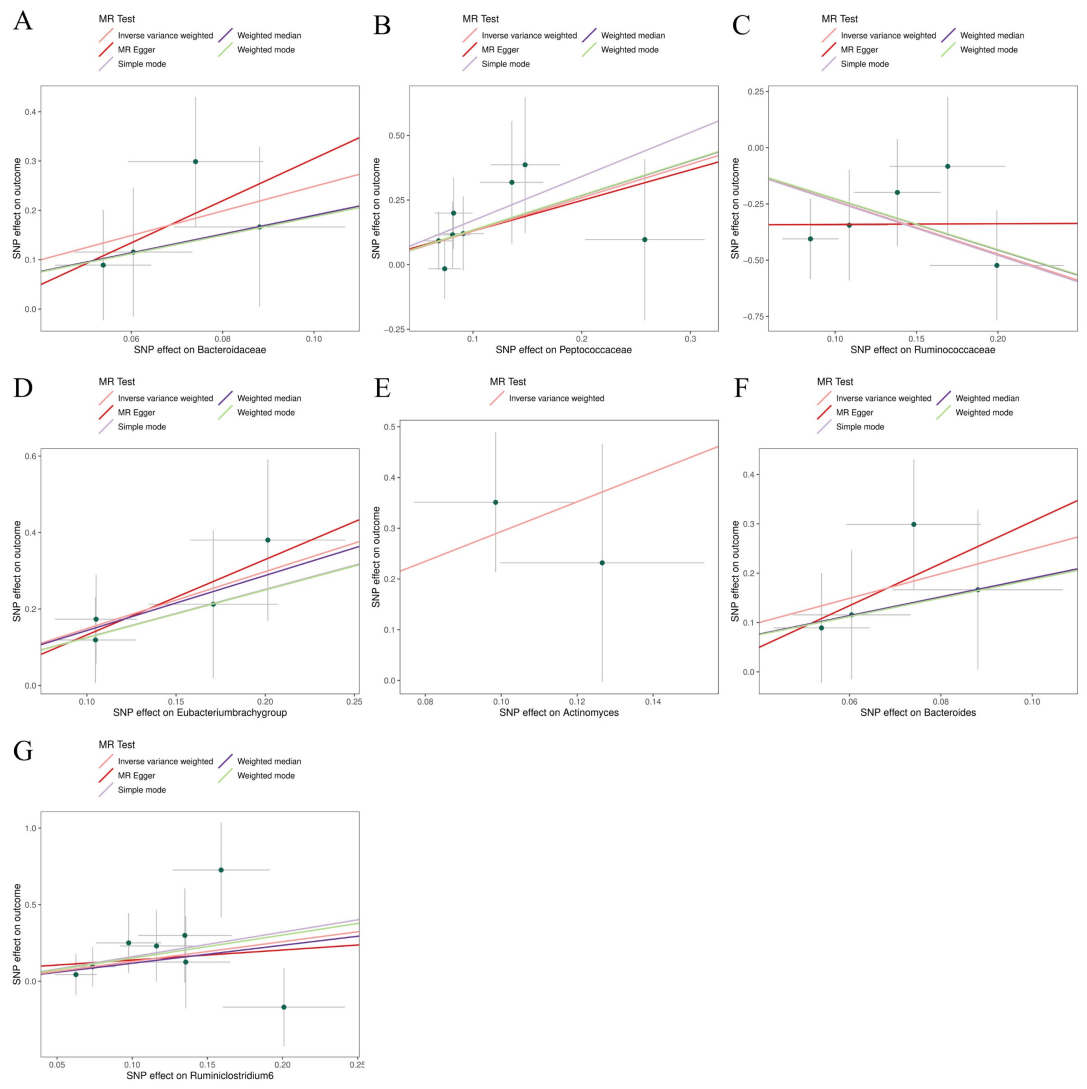
Scatter plots for the causal association between gut microbiota and glioblastoma. (A) Bacteroidaceae; (B) Peptococcaceae; (C) Ruminococcaceae; (D) Eubacterium (brachy group); (E); (F) Bacteroides; (G) Ruminiclostridium6.

**Figure 5:**
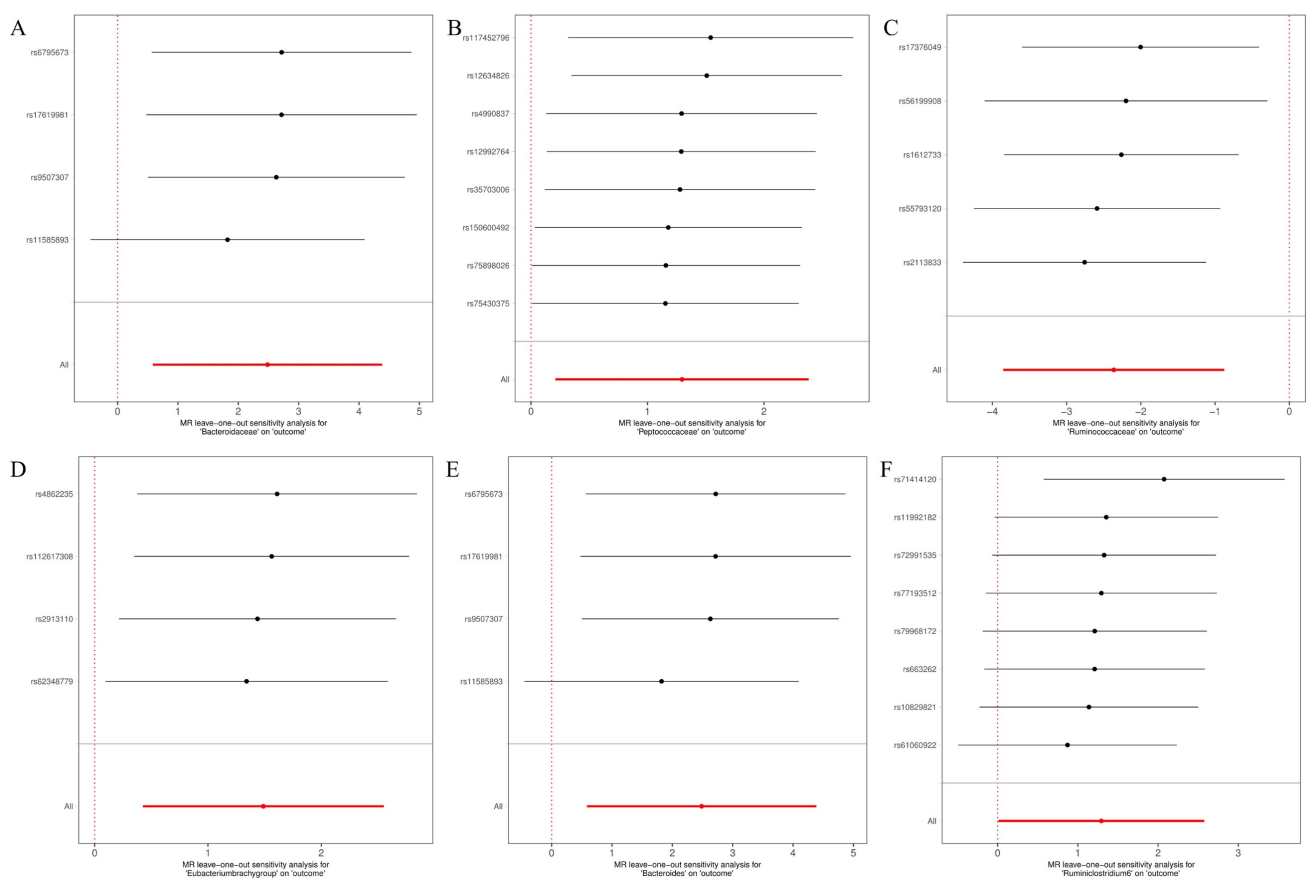
Leave-one-out for the causal association between gut microbiota and glioblastoma. (A) Bacteroidaceae; (B) Peptococcaceae; (C) Ruminococcaceae; (D) Eubacterium (brachy group); (E) Bacteroides; (F) Ruminiclostridium6.

The MR-Egger, weighted mode, simple mode, weighted median, and IVW methods produced comparable causal estimates in both magnitude and direction. Visual inspection revealed probable outliers of the IVs in scatter plots **(Figure 4)** and leave-one-out plots **(Figure 5)**. Our analysis using the MR-Egger regression intercept approach found no indication of horizontal pleiotropy for gut microbiota in glioblastoma, with a p-value greater than 0.05 **(Table S2)**. Results from MR-PRESSO analysis indicated no outliers in the data **(Table S3)**. Moreover, the Cochrane Q statistics results indicated no significant heterogeneity, with a p-value larger than 0.05 **(Table S4)**.

### Bi‑directional causal effects between gut microbiota and glioblastoma risk

We used glioblastoma as exposure and the identified causal bacterial as outcome for evaluating any reverse causation effects. Based on five popular MR methods, we found that glioblastoma was no significance causally associated with the identified causal gut microbiota.

## Discussion

Using a two-sample MR study, we investigated manageable causal relationship between gut microbiota and glioblastoma, with summary statistics for gut microbiota from the International Consortium MiBioGen and summary statistics for glioblastoma from the FinnGen consortium R8 release data (2022). The findings supported the hypothesis that the increase in abundance of genetic susceptibility in the family Ruminococcaceae was once defensive towards glioblastoma, while the different two organizations of the family, Bacteroidaceae and Peptococcaceae, and four microbial genera, namely, Eubacterium (brachy group), Actinomyces, Bacteroides, and Ruminiclostridium 6, had been observed to extend the hazard of glioblastoma with growing heritage susceptibility abundance. With the help of reverse MR analysis, no appreciable causal association between glioblastoma and the identified causal gut microbiota was previously identified.

The causal relationship between hereditary susceptibility of gut microbiota and exceptional cancers has been established, but the additional interest focused on the gut microbiota and gastrointestinal tumors because they are in the same ecosystem and it is less complicated to find a conceivable causal relationship between them 37,38, although the doable causal relationships between gut microbiota and different cancers is constantly mentioned in the literature 39. However, the causal relationship between intestinal plant life and glioblastoma has no longer been reported. Zheng et al. found that composition of the microbiota significantly changed in patients with lung cancer compared with control subjects 40. Zhu et al. also found a change in the gut microbial neighborhood in breast cancer patients 41. It is undeniable that connection between gut microbiota and the development of cancer is receiving more and more attention. However, there is nonetheless a dearth of solid proof on the microbial elements of gut microbiota that make contributions to most cancer development. Although some possible causative linkages between the gut microbiota and cancer have been hypothesized in some animal models due to the complicated interplay between the gut microbiota and the human host, the precise causal relationship between the two remains undetermined. The following limitations apply to observational studies: it is impossible to determine the temporal order between exposure and result, and it is impossible to account for the impact of several confounding variables 42. Gut microbiota is influenced by distinctive factors, including diet 43, BMI 44, medications 45, and different factors 46, all of which contribute to the lack of self-assurance in observational studies. For these reasons, the doable causal relationships between gut microbiota and most cancers nevertheless warrants similar research. Inspired by the application of a massive pattern GWAS database, we have been in a position to use summary-level statistics for causal inference between gut microbiota and glioblastoma, with the hope of exploiting the brain-gut axis for improved interpretation.

A growing body of research found possible links between gut microbiota selected for our study and other cancers. For instance, Patients with intrahepatic cholangiocarcinoma (ICC) had a greater abundance of family Peptostreptococcaceae than in sufferers with hepatocellular carcinoma or cirrhosis and in healthy individuals 47. Abundance of the family of Ruminococcaceae was higher in patients with vascular invasion (VI) than in patients with ICC without VI 48. Our MR results suggetsted that family Peptostreptococcaceae is a risk factor for glioblastoma. In a study of the association between the gut microbiome and primary liver cancer using a two-sample Mendelian randomization and case-control approach, the family Ruminococcaceae was found as a protective factor against hepatocellular liver cancer and the genus Bacteroidetes as a protective factor for intrahepatic cholangiocarcinoma in a two-sample MR study. In contrast, in case-control studies, healthy controls possessed higher relative abundance of the family Ruminococcaceae and the genus Bacteroidetes than patients with hepatocellular hepatocellular carcinoma 49. As shown in our results, our MR results suggested that family Ruminococcaceae was also found as a protective factor against glioblastoma, while the genus Bacteroides and the family Bacteroidaceae are risk factors for glioblastoma. One study showed that genus Ruminiclostridium 6 might be potential pathogens with a low malignant potential in plasmacytoid ovarian cancer 6. In a study of oral microbiota as novel biomarkers for colorectal cancer screening, Eubacterium (brachy group) was ideal for differentiating healthy controls (HCs) from colorectal cancer (CRC) patients 50. And in the present study, we also found Eubacterium (brachy group) to be a risk factor for glioblastoma. The family Actinomycetaceae of the order Actinomycetales, which also includes the families Mycobacteriaceae (Mycobacterium), Nocardiaceae (Nocardia, Rhodococcus), Corynebacteriaceae (Corynebacterium), and others, contains the genus Actinomyces. All belong to the Actinobacteria phylum 51.

Gut microbiota and various intestinal metabolites influence glioma development and progression through neural signaling, microglia regulation, and energy metabolism 52. Gut microbiota is involved in regulation of glioma proliferation and immune response. A study comparing the changes that occur in gut microbiota of glioma-bearing mice compared to healthy mice found a significant decrease in ratio of the Firmicutes to the Bacteroidetes and showed significant differences in relative abundance of the Verrucomicrobia and Akkermansia. This shows that there is a correlation between reduced abundance or structural dysregulation of the bacterial flora and glioma progression 53. The presence of a large number of immune cells and functional lymphatic vessels in the glioma microenvironment and the dysfunction of the lymphatic network constituted by them can promote the progression of glioma 54,55. Since the gut microbiota itself can participate in regulating development and function of immune cells, and its metabolites can also influence function of the lymphatic network, the flora can be directly or indirectly involved in the regulation of glioma progression 56. In addition, dysbiosis of gut microbiota can induce a suppressed immune response in the tumor microenvironment, thus increasing the immune escape of glioma cells and accelerating the progression of glioma 56,57. Loss of flora diversity also leads to a defective immune function in the CNS, which promotes the proliferation of tumor-associated macrophages, mainly abnormal microglia, and ultimately promotes glioma progression 58. Therefore, we can draw the following inference that modulation or transplantation of bacterial flora is expected to be a new means of treatment for glioma by modulating the immune system.

The main gut microbiota of the organism that produce SCFAs are Bacteroides, Bifidobacterium, Propionibacterium, Lactobacillus, Clostridium, Roseburia, and Pseudomonas spp 59. Amongst the seven gut microbiomes we explored in this study, Bacteroidaceae is a family of bacteria in the order Bacteroidetes, and the type genus of this family is Bacteroides 51. In glioma, SCFAs regulate growth and metabolism of glioma cells by affecting immunity, angiogenesis, and epigenetic modifications of the body. In addition to SCFAs, non-SCFAs metabolites produced by gut microbiota metabolism also have a wide range of regulatory effects on organism. Polyamines and nitric oxide are derivatives of spermidine and are also produced by the metabolism of gut microbiota 60. Nitric oxide, on the other hand, promotes tumor cell growth by inhibiting the JAK3-STAT5 signaling pathway, interfering with T cell function and inducing apoptosis 61.

The gut microbiota is involved in the regulation of multiple systems of the body by directly or indirectly influencing hormone secretion and immune response, and is involved in regulating multiple response responses in the glioma microenvironment. In addition, SCFAs and amino acids in the metabolites of the flora are not only involved in the immune response to glioma, but also in the regulation of gene epigenetic modifications. Therefore, gut microbiota and its metabolites can be used as potential targets for anti-glioma therapy, providing ideas and directions for the discovery of new targets for anti-glioma therapy.

There are advantages of this study in the following points: Compared to traditional observational studies, MR analysis can usually achieve RCT-like results that are less subject to confounding factors and reverse causality. Therefore, this study used a two-sample MR framework using genetic variation to assess and analyze causal relationships between gut microbiota and glioblastoma with reverse causal inference. Genetic variation in the gut microbiota was obtained by maximal GWAS meta-analysis, ensuring strength of instrumentation in the MR analysis. Multiple methods were used to perform sensitivity analyses with consistent results, and the robustness of our findings was demonstrated using MR-PRESSO and MR-Egger regression intercept tests to detect and exclude horizontal pleiotropy.

There are certain limitations in this study that need to be noted when we interpret the results. First, GWAS of gut microbiota was obtained from the International Consortium MiBioGen, which included populations from different countries, mainly European populations, while GWAS of GBM was obtained from the FinnGen consortium R8 release data, which included populations of Finnish individuals. Due to the different exposure and outcome GWAS populations, demographic heterogeneity may have biased the results, while the generalizability of MR results in other populations warrants future investigation. Second, the limited number of GBM cases in the FinnGen data and the lack of specific typing of glioblastoma subtypes in the GWAS database may reduce the persuasiveness of this study and lead to a poor use of gut flora to explain the treatment response and prognosis of different subtypes of glioblastoma. Therefore, a further increase in the number of glioblastoma cases and subtyping of glioblastoma is needed to investigate potential causal relationships between gut microbiota and different subtypes of glioblastoma in more depth. Third, we lowered the *P* threshold between exposure and instrumental variables, which may increase the risk of violating the first hypothesis of MR. However, we performed an F-statistic test for each SNP and did not find SNPs with F-statistic values less than 10, indicating absence of weak SNPs in MR estimates. To better investigate disease pathogenesis, recent studies have proposed the use of multiple histological platforms for an integrated understanding analysis of disease pathogenesis in the context of complex interactions of genetic and environmental factors over time 62.

## Conclusions

In summary, our study comprehensively assessed the causal relationship between gut microbiota and glioblastoma. Our results suggest that there are one positive causal direction and six negative directions with glioblastoma. This study may provide new insights into mechanisms and drug-targets of gut microbiota-mediated cancer development.

## Supplementary Material

Supplementary tables.

## Figures and Tables

**Table 1:** MR results of causal effects between gut microbiota and glioblastoma (*P*<5×10^-6^)

Gut microbiota(exposure)	method	nSNP	*β*	SE	p-value	OR	95%CI
Family Bacteroidaceae	MR Egger	4	4.27	5.26	0.50	71.39	0.00-2145757
	WME	4	1.90	1.13	0.09	6.68	0.73-61.61
	IVW	4	2.46	0.97	0.01	12.00	1.79-80.32
	Simple mode	4	1.87	1.61	0.33	6.49	0.28-151.74
	Weighted mode	4	1.87	1.57	0.32	6.49	0.30-140.86
Family Peptococcaceae	MR Egger	8	1.18	1.51	0.46	3.27	0.17-62.92
	WME	8	1.34	0.75	0.07	3.82	0.88-16.46
	IVW	8	1.30	0.55	0.02	3.66	1.23-10.84
	Simple mode	8	1.71	1.15	0.18	5.51	0.58-52.60
	Weighted mode	8	1.34	1.04	0.24	3.83	0.50-29.20
Family Ruminococcaceae	MR Egger	5	0.03	2.43	0.99	1.03	0.00-119.62
	WME	5	-2.27	1.02	0.03	0.10	0.01-0.76
	IVW	5	-2.36	0.76	<0.01	0.09	0.02-0.42
	Simple mode	5	-2.39	1.40	0.16	0.09	0.00-1.43
	Weighted mode	5	-2.27	1.31	0.16	0.10	0.00-1.36
Genus Eubacteriumbrachygroup	MR Egger	4	1.97	2.00	0.43	7.18	0.14-361.09
	WME	4	1.44	0.67	0.032	4.21	1.13-15.67
	IVW	4	1.49	0.54	<0.01	4.43	1.53-12.84
	Simple mode	4	1.26	0.84	0.23	3.52	0.68-18.09
	Weighted mode	4	1.25	0.86	0.24	3.49	0.64-18.95
Genus Actinomyces	IVW	2	2.93	1.11	<0.01	18.80	2.12-167.17
Genus Bacteroides	MR Egger	4	4.27	5.26	0.50	71.39	0.00-2145757
	WME	4	1.90	1.16	0.10	6.68	0.69-65.18
	IVW	4	2.49	0.97	0.01	12.00	1.79-80.32
	Simple mode	4	1.87	1.59	0.32	6.49	0.29-147.15
	Weighted mode	4	1.87	1.56	0.32	6.49	0.31-137.60
Genus Ruminiclostridium6	MR Egger	8	0.65	1.75	0.72	1.92	0.06-59.16
	WME	8	1.18	0.90	0.19	3.24	0.56-18.78
	IVW	8	1.29	0.65	0.05	3.64	1.01-13.14
	Simple mode	8	1.60	1.59	0.35	4.98	0.22-111.38
	Weighted mode	8	1.51	1.39	0.31	4.52	0.30-69.10

CI, confidence interval; IVW, Inverse variance weighted; MR, Mendelian randomization; SNP, single nucleotide polymorphism; SE, standard error; OR, Odds ratio; WME, weighted median estimator.

## Abbreviations

AA: acetic acid
BA: butyric acid
CRC: colorectal cancer
MR: Mendelian randomization
MAF: minor allele frequency
HC: healthy control
IVW: Inverse variance weighted
IV: instrumental variable
ICC: intrahepatic cholangiocarcinoma
IGF-1: insulin-like growth factor-1
LD: linkage disequilibrium
PA: propionic acid
SNP: single nucleotide polymorphism
SCFAs: short-chain fatty acids
VI: vascular invasion
WME: weighted median estimator
