# Supplementary material for: Causal relationship between gut microbiota and glioblastoma: a two-sample Mendelian randomization study
Source: J Cancer. 2024 Jan 1;15(2):332–42. doi: 10.7150/jca.90149 (PMC10758031; doi:10.7150/jca.90149)
Supplement: Supplementary file 1 — Supplementary tables. [file jcav15p0332s1.pdf]

Supplementary Material

Table S1. Instrumental variables used in MR analysis of the association between gut microbiota and glioblastoma.

id.exposure	SNP	Effect allele	Other allele	Outcome			Exposure			eaf. exposure	F
				beta	se	pval	beta	se	pval		
Family Bacteroidaceae	rs11585893	A	G	-0.30	0.13	0.02	-0.07	0.01	1.80E-06	0.15	25.17
Family Bacteroidaceae	rs17619981	T	G	0.17	0.16	0.30	0.09	0.02	2.69E-06	0.07	22.19
Family Bacteroidaceae	rs6795673	C	T	0.09	0.11	0.42	0.05	0.01	3.38E-07	0.41	26.18
Family Bacteroidaceae	rs9507307	C	T	0.12	0.13	0.38	0.06	0.01	2.13E-06	0.19	21.91
Family Peptococcaceae	rs117452796	A	G	-0.10	0.31	0.76	-0.26	0.05	3.15E-06	0.07	22.03
Family Peptococcaceae	rs12634826	T	G	0.02	0.12	0.89	-0.07	0.02	1.01E-06	0.30	24.03
Family Peptococcaceae	rs12992764	T	G	0.09	0.11	0.42	0.07	0.01	1.46E-06	0.40	23.50
Family Peptococcaceae	rs150600492	A	C	0.32	0.24	0.18	0.14	0.03	2.31E-06	0.06	21.95
Family Peptococcaceae	rs35703006	G	T	0.12	0.13	0.37	0.08	0.02	4.95E-07	0.22	24.45
Family Peptococcaceae	rs4990837	A	G	0.12	0.14	0.40	0.09	0.02	1.74E-06	0.17	23.99
Family Peptococcaceae	rs75430375	C	T	-0.39	0.26	0.14	-0.15	0.03	3.41E-06	0.06	21.71
Family Peptococcaceae	rs75898026	A	G	-0.20	0.14	0.15	-0.08	0.02	2.02E-06	0.18	22.42
Family Ruminococcaceae	rs1612733	T	C	-0.34	0.25	0.16	0.11	0.02	4.22E-06	0.06	20.86
Family Ruminococcaceae	rs17376049	T	C	-0.41	0.178	0.02	0.08	0.02	7.30E-07	0.10	24.30
Family Ruminococcaceae	rs2113833	T	C	-0.08	0.31	0.79	0.17	0.04	1.14E-06	0.04	22.70
Family Ruminococcaceae	rs55793120	T	C	-0.20	0.24	0.40	0.14	0.03	1.44E-07	0.05	26.97
Family Ruminococcaceae	rs56199908	T	C	0.52	0.24	0.03	-0.20	0.04	1.66E-06	0.05	23.58
Genus Eubacteriumbrachygroup	rs112617308	T	C	-0.21	0.19	0.27	-0.17	0.04	2.38E-06	0.08	22.17
Genus Eubacteriumbrachygroup	rs2913110	C	T	0.17	0.12	0.14	0.11	0.02	4.56E-06	0.38	21.00
Genus Eubacteriumbrachygroup	rs4862235	G	A	0.12	0.11	0.29	0.10	0.02	3.73E-06	0.44	21.55
Genus Eubacteriumbrachygroup	rs62348779	T	C	-0.38	0.21	0.07	-0.20	0.04	3.78E-06	0.08	21.66
genus.Actinomyces	rs34583783	G	T	0.23	0.23	0.32	0.13	0.03	4.49E-06	0.10	22.23
Genus Actinomyces	rs4146653	G	A	0.35	0.14	0.01	0.10	0.02	4.50E-06	0.20	21.16
Genus Bacteroides	rs11585893	A	G	-0.30	0.13	0.02	-0.07	0.01	1.80E-06	0.15	25.17
Genus Bacteroides	rs17619981	T	G	0.17	0.16	0.30	0.09	0.02	2.69E-06	0.07	22.19
Genus Bacteroides	rs6795673	C	T	0.09	0.11	0.42	0.05	0.01	3.38E-07	0.41	26.18
Genus Bacteroides	rs9507307	C	T	0.12	0.13	0.38	0.06	0.01	2.13E-06	0.19	21.91
Genus Ruminiclostridium6	rs10829821	T	C	-0.25	0.19	0.20	-0.10	0.02	3.47E-06	0.09	20.40

Genus Ruminiclostridium6	rs11992182	A	C	0.04	0.13	0.75	0.06	0.01	4.65E-06	0.29	20.57
Genus Ruminiclostridium6	rs61060922	T	G	0.73	0.31	0.02	0.16	0.03	1.09E-06	0.04	24.38
Genus Ruminiclostridium6	rs663262	T	C	-0.30	0.31	0.33	-0.13	0.03	3.39E-06	0.05	18.87
Genus Ruminiclostridium6	rs71414120	T	G	-0.17	0.25	0.50	0.20	0.04	1.08E-06	0.08	24.43
Genus Ruminiclostridium6	rs72991535	T	G	0.13	0.30	0.68	0.14	0.03	4.95E-06	0.06	21.10
Genus Ruminiclostridium6	rs77193512	A	G	0.09	0.13	0.46	0.07	0.02	1.30E-06	0.17	23.12
Genus Ruminiclostridium6	rs79968172	G	A	0.23	0.23	0.32	0.11	0.02	1.66E-06	0.06	22.82

MR, Mendelian randomization; SNP, single nucleotide polymorphism; eaf, effect allele frequency; SE, standard error.

Table S2. Directional horizontal pleiotropy assessed by intercept term in MR Egger regression of the association between gut microbiota and glioblastoma.

id.exposure	Egger_intercept	se	pval
Family Bacteroidaceae	-0.12	0.35	0.76
Family Peptococcaceae	0.01	0.14	0.94
Family Ruminococcaceae	-0.34	0.33	0.37
Genus Eubacteriumbrachygroup	-0.06	0.26	0.83
Genus Actinomyces	NA	NA	NA
Genus Bacteroides	-0.12	0.35	0.76
Genus Ruminiclostridium6	0.07	0.18	0.71

MR, Mendelian randomization; se, standard error.

Table S3. The heterogeneity of gut microbiota instrumental variables.

id.exposure	method	Q	Q_df	Q_pval
Family Bacteroidaceae	MR Egger	0.98	2	0.61
Family Bacteroidaceae	Inverse variance weighted	1.10	3	0.78
Family Peptococcaceae	MR Egger	2.86	6	0.83
Family Peptococcaceae	Inverse variance weighted	2.86	7	0.90
Family Ruminococcaceae	MR Egger	1.75	3	0.63
Family Ruminococcaceae	Inverse variance weighted	2.83	4	0.59
Genus Eubacteriumbrachygroup	MR Egger	0.26	2	0.88
Genus Eubacteriumbrachygroup	Inverse variance weighted	0.32	3	0.96
Genus Actinomyces	Inverse variance weighted	0.56	1	0.45
Genus Bacteroides	MR Egger	0.98	2	0.61
Genus Bacteroides	Inverse variance weighted	1.10	3	0.78
Genus Ruminiclostridium6	MR Egger	6.33	6	0.39
Genus Ruminiclostridium6	Inverse variance weighted	6.50	7	0.48

Df, degree of freedom.

Table S4. MR-PRESSO analysis for the association between gut microbiota and glioblastoma.

Exposure	MR Analysis	b	se	T-stat	pval	Global_Test RSSobs	Global_Test Pvalue	OR (95% CI)	P value
Family Bacteroidaceae	MR-PRESSO	2.49	0.59	4.23	0.02	2.14	0.77	12.00 (3.79~38.01)	0.024
Family Peptococcaceae	MR-PRESSO	1.30	0.35	3.65	0.01	3.82	0.90	3.66 (1.82~7.33)	<0.01
Family Ruminococcaceae	MR-PRESSO	-2.36	0.64	-3.70	0.02	4.01	0.68	0.09 (0.03~0.33)	0.021
Genus Bacteroides	MR-PRESSO	2.49	0.59	4.23	0.02	2.14	0.79	12.00 (3.79~38.01)	0.024
Genus Eubacteriumbrachygroup	MR-PRESSO	1.49	0.18	8.36	<0.01	0.59	0.96	4.43 (3.13~6.28)	<0.01
Genus Ruminiclostridium6	MR-PRESSO	1.29	0.63	2.05	0.08	9.94	0.42	3.64 (1.06~12.54)	0.08

MR, Mendelian randomization; se, standard error; RSS_{obs}, observed residual sum of squares.
